# Supplementary material for: Utility of carbon and nitrogen stable isotopes for inferring wild bee (Hymenoptera: Apoidea) use of adjacent foraging habitats
Source: PLoS One. 2022 Jul 13;17(7):e0271095. doi: 10.1371/journal.pone.0271095 (PMC9278760; doi:10.1371/journal.pone.0271095)
Supplement: S1 File — (DOCX) [file pone.0271095.s002.docx]

**Utility of carbon and nitrogen stable isotopes for inferring wild bee (Hymenoptera: Apoidea) use of adjacent foraging habitats**

Jessie Lanterman Novotny^1^ and Karen Goodell^2^

^1^ Hiram College, Department of Biology

^2^ The Ohio State University, Department of Evolution, Ecology, and Organismal Biology

**Supporting Information**

***Results of preliminary laboratory feeding trial***

A laboratory feeding trial was not conducted due to the difficulty of obtaining bee eggs and large quantities of pollen to rear them on exclusively from plants grown in the edge, forest, or field habitat. However, a preliminary study was conducted to determine whether carbon from the adult nectar diet gets incorporated into bee tissues. Wild-caught adult bumble bee workers were housed individually and fed either a control diet of beet sugar syrup (C3 plant, ∂^13^C = -26.68) or a treatment diet of cane sugar syrup (C4 plant, ∂^13^C = -11.96) (Goodell & Frankenberry, *unpubl. data*). All bees were assumed to have been reared as larvae on pollen and nectar from plants that fix C3 carbon during photosynthesis (similar to beets), which make up the majority of bee pollinated plants in the study region. After 10 days of consuming a controlled diet of sugar water in captivity, bees were euthanized and prepared for isotope analysis. Adult workers fed the cane sugar diet became significantly lighter in ∂^13^C during the study period than those fed beet sugar (flight muscle cane versus beet sugar *F*_1,16_ = 55.64, *p* < 0.01; abdomen chitin *F*_1,16_ = 98.81, *p* < 0.01). The difference was apparent in both tissue types, but even more pronounced in the abdomen chitin than in the flight muscle (abdomen chitin beet sugar ∂^13^C = -26.82 ± 0.24, n = 10; cane sugar ∂^13^C = -20.83 ± 1.90, n = 8). These results suggest that bees incorporate C from their adult nectar diet relatively rapidly, especially in the abdomen. Therefore, field-caught wild bees should reflect differences in C isotope signature of their food, given there are underlying spatial and temporal differences in plant isotope composition.

***Results of Bee Head+Thorax (HT) Isotope Analysis***

There were small, but significant differences in ∂^13^C isotope composition of the two bee tissue types we tested. Head+thorax samples (hereafter referred to as “HT”) had lower and less variable lipid content than abdomens according to their C:N ratios (an established proxy for lipid content in animals; McConnaughey and McRoy 1979, Post et al. 2007; t = 8.89, df = 90.93, p < 0.01, abdomen 6.04 ± 2.03 SD, HT 4.12 ± 0.21). After ∂^13^C was lipid normalized (McConnaughey and McRoy 1979), abdomen samples were significantly heavier (less negative) in ∂^13^C than HT (t = 3.84, df = 173.60, p < 0.01, abdomen mean ∂^13^C_lipidnormalized_ -25.58 ± 0.69 SD, headthorax -26.01 ± 0.78). There was no difference in ∂^15^N between HT and abdomen tissues (*F*_1,176_ = 0.02, *p* = 0.88). Despite differences in ∂^13^C, the two tissues showed similar trends by habitat, month, and taxonomic and social group.

As with the abdomens, HT ∂^13^C_lipid normalized_ was significantly influenced by genuscaste and ∂^15^N by habitat and by genuscaste (**Table S5**). However, HT ∂^13^C_lipid normalized_ was marginally but not significantly influenced by habitat in multiple linear models (p = 0.06). Similar to the abdomens, bees collected in the field were depleted in HT ∂^13^C and in ∂^15^N relative to those caught along the edge (Tukey-adjusted model pairwise contrasts ∂^13^C *t* = -1.90, df = 83, *p* = 0.06; ∂^15^N *t* = -2.53, df = 83, *p* = 0.0; **Fig S1**). According to model contrasts of geunscaste groups, *Halictus* HT samples were depleted in ∂^13^C_lipid normalized_ compared to *Bombus* and *Andrena.* *Andrena* were not distinguishable from *Halictus* in ∂^15^N. Bumble bees were enriched in HT ∂^15^N compared to the other genera, possibly due to differences in larval feeding in *Bombus* colonies. Overwintered spring *Bombus* queens were enriched in HT ∂^13^C compared to workers, but not distinct from summer queens-of-the-year, possibly a signature of starvation during overwintering. *Bombus* summer queens-of-the-year were enriched in ∂^15^N compared to overwintered spring queens and workers, which may indicate intense N demand needed to mature sex organs after mating.

It should be noted that individuals showed little variability within a genus in HT ∂^13^C_lipid normalized_ (*Bombus* coefficient of variation CV = 2.23%, *Andrena* CV = 4.17%, and *Halictus* CV = 1.90%). However, *Andrena* and *Halictus* HT samples showed greater variability in ∂^15^N than those of bumble bees (*Andrena CV* = 160.58%, *Halictus CV* = 172.12%, *Bombus CV* = 48.13%), possibly due to differences between taxa in brood provisioning versus larval feeding.

Seasonal trends were analyzed using a subset of data from one genuscaste group that was represented in all months (*Bombus* workers), because in the full dataset month and genus were conflated due to inherent differences between taxa in the timing of their annual activity. *Bombus* workers were enriched in ∂^13^C_lipid normalized_ in July compared to August (*F*_2,44_ = 3.75, *p* = 0.03; **Fig S2**). Worker ∂^15^N was greater in July than in June and August (*F*_2,44_ = 5.600, *p* = 0.006).

Isotope mixing models (created in MixSIAR using the “long” run settings) attributed a smaller proportion than expected of forest flowers to *Andrena* HT isotope composition, given that most species are active only in the spring and early summer (**Table S6**). Conversely, models estimated that over half of a summer flying sweat bee’s (*Halictus*) diet came from forest resources. In overwintered spring *Bombus* queens, the mixing model predicted that half of their diet came from field flowers, even though queens are known to forage on forest understory and edge plants in spring. *Bombus* summer queens-of-the-year and workers were indistinguishable in diet contribution, with the majority of their predicted diet coming from edge habitat. This is not surprising given their relatively long period of worker activity and far flight range. There are several possible explanations for the seemingly counterintuitive mixing model results for *Andrena*, *Halictus*, and *Bombus* overwintered spring queens that seem to contradict typical foraging habitats of adults. The first possible explanation is that the mixing models did not resolve bees diets well because of high overlap and variability in potential food sources from the forest, edge, and field. A second explanation, however, is that these results are in fact ecological valuable, but are confounded by the mismatch in timing and location of food collected to feed the larvae versus adults. For example, *Bombus* workers active in the summer collect pollen and nectar to feed larvae from field and edge flowers (based on the availability of food in the landscape at that time in this study region). Some of those larvae turn into new queens that will feed on field flowers to create fat stores, mate, then overwinter. Those queens chitin, muscle, and fat reserves should then reflect the field flowers they were fed as larvae. When they emerge the following spring, they forage primarily on forest and edge plants during the nest founding period. Therefore, the abdomen tissue may more strongly resemble plants from a different habitat than HT tissue. This mismatch between the resources used in larval development to create the chitin and flight muscles in the HT (from the field in the case of *Bombus* queens) and the resources used in adulthood to fuel current metabolic needs and oviposition (forest/edge) is a barrier to using stable isotopes to understand spatial foraging patterns.

***List of Supporting Information Tables and Figures***

**Table S1. Bee and flower C and N stable isotope raw data from this study** [separate .csv file not included here]

**Table S2**. Mean ∂^13^C and ∂^15^N values of bee and flower samples. The total number of samples of each tissue type is given as *n*. Bee ∂^13^C values were lipid normalized following McConnaughey and McRoy (1979).

**Table S3. Pairwise contrasts among levels of bee abdomen model predictors.** Tukey-adjusted pairwise comparisons were calculated using R function *lsmeans*. Significant contrasts (*p* < 0.05) are indicated in bold.

**Table S4. Pairwise contrasts among levels of flower model predictor variables.** Tukey-adjusted pairwise comparisons were calculated using R function *lsmeans*. Significant contrasts (*p* < 0.05) are indicated in bold.

**Table S5**. **The effects** **of habitat, genus, and social caste on wild bee headthorax C and N isotope composition**. Here we give the overall test statistic for each predictor and associated p values from model ANOVAs computed using Type III (simultaneous) sum of squares.

**Table S6**. **Predicted proportion diet contributions of field, edge, and forest flowers to bee headthorax C and N isotope composition**, estimated from isotope mixing models created in MixSIAR. ∂^13^C values were lipid normalized following the methods of McConnaughey and McRoy (1979). Mean diet contributions ± SD, with median, lower, and upper 95% Bayesian credible intervals are given.

**Fig S1. Habitat trends in natural abundances of C and N** **stable isotopes** **in bee head+thorax and flowers: (a) bee ∂^13^C_lipid normalized_, (b) flower ∂^13^C, (c) bee ∂^15^N, (d) flower ∂^15^N.** The center line represents group median, with boxes showing upper and lower quartiles. The bars show value range. Bee sample sizes were as follows: field n=25, edge n=64, forest n=0. Flower sample sizes were as follows: field n=10, edge n=16, forest n=6.

**Fig S2**. **Bee head+thorax isotope composition by taxonomic and social caste groups**.

(a) *Andrena carlini* (AprMay) are shown as dark blue squares, *A. crateaegi* (Jun) as light blue. (b) *Halictus ligatus* (Jun – Aug) are shown as dark purple squares, *H. confusus* (Jul) as light purple, (c) *Bombus* overwintered spring queens (AprMay) are shown as white squares, summer queens-of-the-year (Jul – Aug) as black, and summer workers (Jun – Aug) as gray. Bombus isotope values did not differ significantly by species. Bee ∂^13^C was lipid normalized following (McConnaughey and McRoy (1979).

**Fig S3. Seasonal trends in natural abundances of C and N** **stable isotopes** **in bee head+thorax and flowers: (a) bee ∂^13^C_lipid normalized_, (b) flower ∂^13^C, (c) bee ∂^15^N, (d) flower ∂^15^N.** Samples collected at the end of April were combined with those from the beginning of May. The center line represents group median, with boxes showing upper and lower quartiles. The bars show value range. Bee sample sizes were as follows: AprMay n=21, June n=20 , July n=32, August n=16. Flower sample sizes were as follows: AprMay n=15, June n=5, July n=9, August n=3.

**Table S2**. Mean ∂^13^C and ∂^15^N values of bee and flower samples. The total number of samples of each tissue type is given as *n*. Bee ∂^13^C values were lipid normalized following McConnaughey and McRoy (1979).

| **Tissue type** | **Isotope** | **Mean** | **SD** | **Min** | **Max** | **n** |
| --- | --- | --- | --- | --- | --- | --- |
| bee abdomen | ∂^13^C | -26.89 | 0.88 | -28.78 | -25.12 | 89 |
|  | ∂^13^C_lipid normalized_ | -25.58 | 0.69 | -27.64 | -24.01 | 89 |
|  | ∂^15^N | 1.28 | 1.86 | -3.76 | 5.07 | 89 |
| bee head+thorax | ∂^13^C | -26.13 | 0.75 | -28.36 | -24.18 | 89 |
|  | ∂^13^C_lipid normalized_ | -26.01 | 0.78 | -28.50 | -24.37 | 89 |
|  | ∂^15^N | 1.32 | 1.91 | -4.30 | 4.94 | 89 |
| flower | ∂^13^C | -28.58 | 1.65 | -31.27 | -25.73 | 32 |
|  | ∂^15^N | -2.01 | 1.44 | -5.19 | 0.22 | 32 |

**Table S3. Pairwise contrasts among levels of bee abdomen model predictors.** Tukey-adjusted pairwise comparisons were calculated using R function *lsmeans*. Significant contrasts (*p* < 0.05) are indicated in bold.

| **isotope** | **parameter** | **contrast** | **estimate** | **SE** | **df** | ***t*** | ***p*** |
| --- | --- | --- | --- | --- | --- | --- | --- |
| ∂^13^C_lipid normalized_ | habitat | **edge - field** | 0.556 | 0.196 | 83 | 2.833 | **0.006** |
|  | Genus-caste | **Andrena - BombusQ1** | -1.399 | 0.371 | 83 | -3.773 | **0.003** |
|  |  | Andrena - BombusQ2 | -0.331 | 0.311 | 83 | -1.066 | 0.823 |
|  |  | Andrena - BombusW | 0.257 | 0.175 | 83 | 1.471 | 0.584 |
|  |  | Andrena - Halictus | -0.091 | 0.266 | 83 | -0.341 | 0.997 |
|  |  | **BombusQ1 - BombusQ2** | 1.068 | 0.369 | 83 | 2.897 | 0.**038** |
|  |  | **BombusQ1 - BombusW** | 1.657 | 0.337 | 83 | 4.915 | **<0.001** |
|  |  | **BombusQ1 - Halictus** | 1.309 | 0.338 | 83 | 3.875 | **0.002** |
|  |  | BombusQ2 - BombusW | 0.589 | 0.276 | 83 | 2.137 | 0.215 |
|  |  | BombusQ2 - Halictus | 0.241 | 0.296 | 83 | 0.812 | 0.926 |
|  |  | BombusW - Halictus | -0.348 | 0.226 | 83 | -1.544 | 0.537 |
| ∂^15^N | habitat | **edge - field** | 1.140 | 0.405 | 83 | 2.820 | **0.006** |
|  | Genus-caste | **Andrena - BombusQ1** | -2.277 | 0.766 | 83 | -2.974 | **0.031** |
|  |  | **Andrena - BombusQ2** | -4.178 | 0.642 | 83 | -6.512 | **<0.001** |
|  |  | **Andrena - BombusW** | -2.108 | 0.361 | 83 | -5.834 | **<0.001** |
|  |  | Andrena - Halictus | 0.427 | 0.550 | 83 | 0.777 | 0.937 |
|  |  | BombusQ1 - BombusQ2 | -1.900 | 0.761 | 83 | -2.496 | 0.101 |
|  |  | BombusQ1 - BombusW | 0.169 | 0.696 | 83 | 0.243 | 0.999 |
|  |  | **BombusQ1 - Halictus** | 2.705 | 0.697 | 83 | 3.878 | **0.002** |
|  |  | **BombusQ2 - BombusW** | 2.070 | 0.569 | 83 | 3.638 | **0.004** |
|  |  | **BombusQ2 - Halictus** | 4.605 | 0.612 | 83 | 7.530 | **<0.001** |
|  |  | **BombusW - Halictus** | 2.535 | 0.466 | 83 | 5.444 | **<0.001** |

**Table S4. Pairwise contrasts among levels of flower model predictor variables.** Tukey-adjusted pairwise comparisons were calculated using R function *lsmeans*. Significant contrasts (*p* < 0.05) are indicated in bold.

| **isotope** | **parameter** | **contrast** | **estimate** | **SE** | **df** | ***t*** | ***p*** |
| --- | --- | --- | --- | --- | --- | --- | --- |
| ∂^13^C | habitat | edge – field | -0.691 | 0.553 | 25 | -1.249 | 0.436 |
|  |  | edge – forest | -0.317 | 0.943 | 25 | -0.336 | 0.940 |
|  |  | field – forest | 0.374 | 0.996 | 25 | 0.376 | 0.925 |
|  | month | AprMay – Aug | -2.306 | 1.084 | 25 | -2.127 | 0.172 |
|  |  | **AprMay – July** | -2.517 | 0.874 | 25 | -2.878 | **0.038** |
|  |  | AprMay – June | -1.791 | 0.956 | 25 | -1.873 | 0.265 |
|  |  | Aug – July | -0.210 | 0.881 | 25 | -0.239 | 0.995 |
|  |  | Aug – June | 0.515 | 0.967 | 25 | 0.533 | 0.950 |
|  |  | July – June | 0.725 | 0.730 | 25 | 0.993 | 0.755 |
|  | growth form | **herb – woody** | -2.744 | 0.930 | 25 | -2.951 | **0.007** |
| ∂^15^N | habitat | edge – field | 1.331 | 0.546 | 25 | 2.437 | 0.056 |
|  |  | edge – forest | 1.295 | 0.931 | 25 | 1.391 | 0.360 |
|  |  | field – forest | -0.035 | 0.983 | 25 | -0.036 | 0.999 |
|  | month | AprMay – Aug | 1.796 | 1.070 | 25 | 1.680 | 0.355 |
|  |  | AprMay – July | 0.990 | 0.863 | 25 | 1.147 | 0.664 |
|  |  | AprMay – June | 0.313 | 0.943 | 25 | 0.331 | 0.987 |
|  |  | Aug – July | -0.807 | 0.869 | 25 | -0.928 | 0.790 |
|  |  | Aug – June | -1.484 | 0.954 | 25 | -1.556 | 0.421 |
|  |  | July – June | -0.677 | 0.720 | 25 | -0.940 | 0.784 |
|  | growth form | herb – woody | 0.295 | 0.917 | 25 | 0.322 | 0.750 |

**Table S5**. **The effects** **of habitat, genus, and social caste on wild bee headthorax C and N isotope composition**. Here we give the overall test statistic for each predictor and associated p values from model ANOVAs computed using Type III (simultaneous) sum of squares.

|  |  | **∂^13^C_lipidnormalized_** **bee HT**  **(R^2^ adj. = 0.254)** | | **∂^15^N bee HT**  **(R^2^ adj. = 0.533)** | |
| --- | --- | --- | --- | --- | --- |
| **Parameter** | **df** | ***F*** | ***p*** | ***F*** | ***p*** |
| Habitat | 1 | 3.604 | 0.061 | 6.377 | 0.013 |
| GenusCaste | 4 | 7.659 | <0.001 | 24.578 | <0.001 |

**Table S6**. **Predicted proportion diet contributions of field, edge, and forest flowers to bee headthorax C and N isotope composition**, estimated from isotope mixing models created in MixSIAR. ∂^13^C values were lipid normalized following the methods of McConnaughey and McRoy (1979). Mean diet contributions ± SD, with median, lower, and upper 95% Bayesian credible intervals are given.

| **Bee Genus/Caste** | **Food Source** | **Mean Diet Contribution** | **Lower** | **Upper** |
| --- | --- | --- | --- | --- |
| *Andrena* females | field flowers | 0.463 ± 0.195 | 0.206 | 0.707 |
|  | edge flowers | 0.376 ± 0.116 | 0.166 | 0.621 |
|  | forest flowers | 0.161 ± 0.087 | 0.030 | 0.363 |
| *Halictus* females | field flowers | 0.318 ± 0.154 | 0.028 | 0.775 |
|  | edge flowers | 0.116 ± 0.086 | 0.016 | 0.338 |
|  | forest flowers | 0.566 ± 0.188 | 0.124 | 0.900 |
| *Bombus*  spring queens | field flowers | 0.548 ± 0.215 | 0.117 | 0.923 |
|  | edge flowers | 0.383 ± 0.214 | 0.036 | 0.821 |
|  | forest flowers | 0.069 ± 0.068 | 0.005 | 0.240 |
| *Bombus* workers | field flowers | 0.110 ± 0.072 | 0.014 | 0.287 |
|  | edge flowers | 0.829 ± 0.088 | 0.638 | 0.961 |
|  | forest flowers | 0.061 ± 0.053 | 0.004 | 0.195 |
| *Bombus* summer queens | field flowers | 0.094 ± 0.074 | 0.010 | 0.289 |
|  | edge flowers | 0.858 ± 0.095 | 0.626 | 0.975 |
|  | forest flowers | 0.048 ± 0.054 | 0.003 | 0.173 |

**Fig S1. Habitat trends in natural abundances of C and N** **stable isotopes** **in bee head+thorax and flowers: (a) bee ∂^13^C_lipid normalized_, (b) flower ∂^13^C, (c) bee ∂^15^N, (d) flower ∂^15^N.** The center line represents group median, with boxes showing upper and lower quartiles. The bars show value range. Bee sample sizes were as follows: field n=25, edge n=64, forest n=0. Flower sample sizes were as follows: field n=10, edge n=16, forest n=6.

Habitat


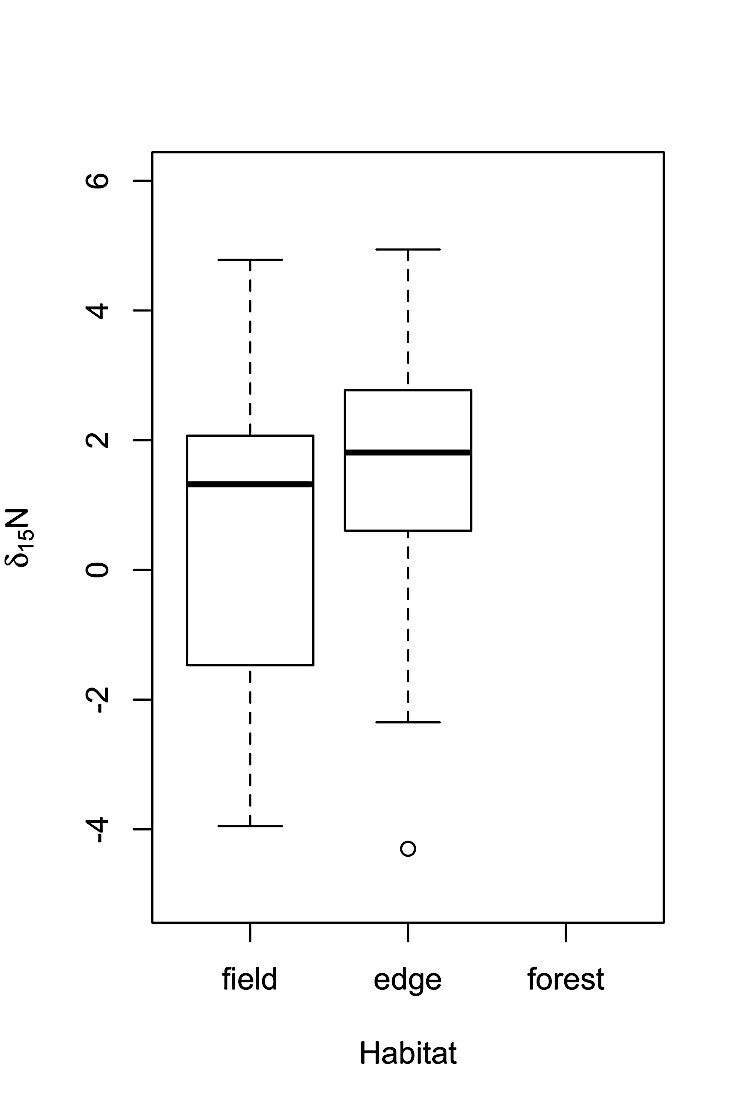

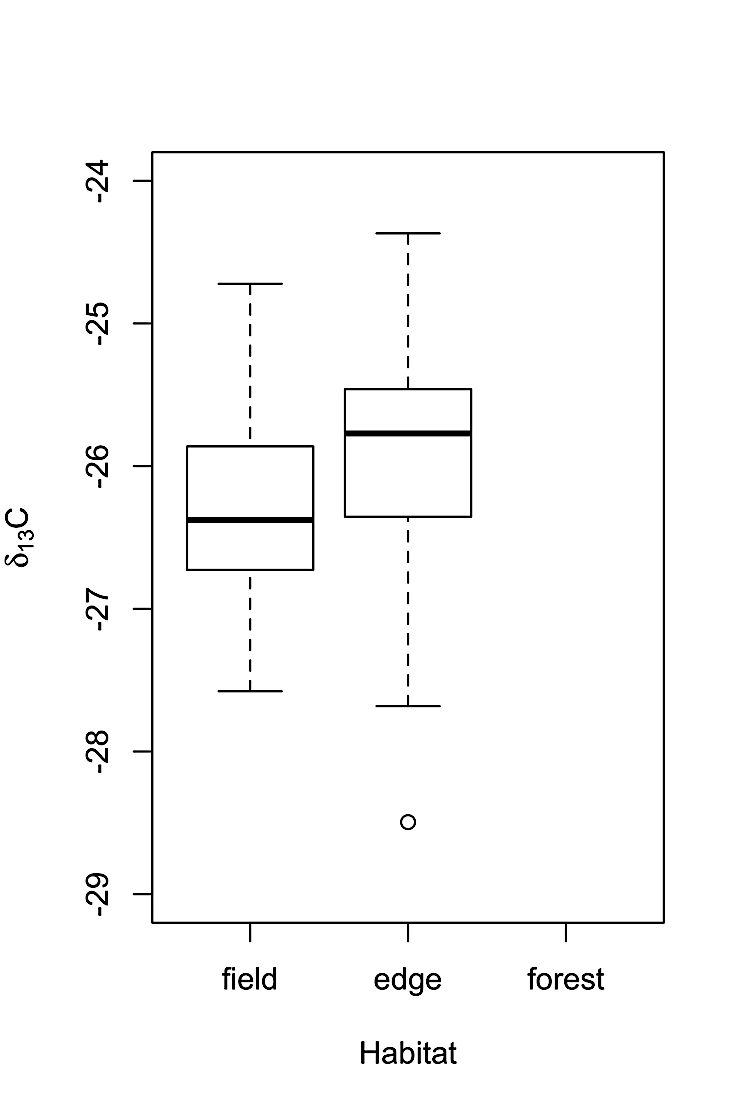

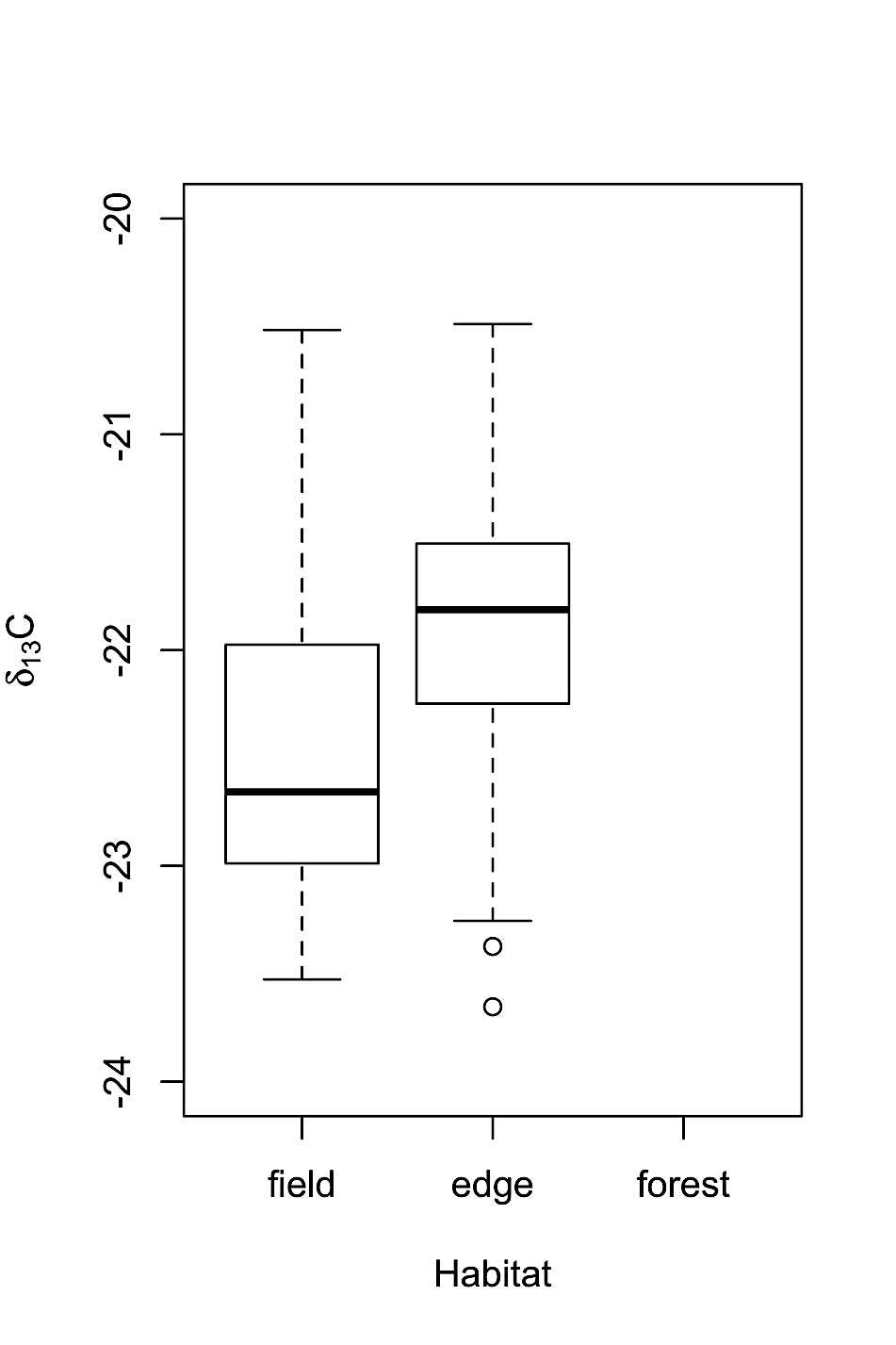

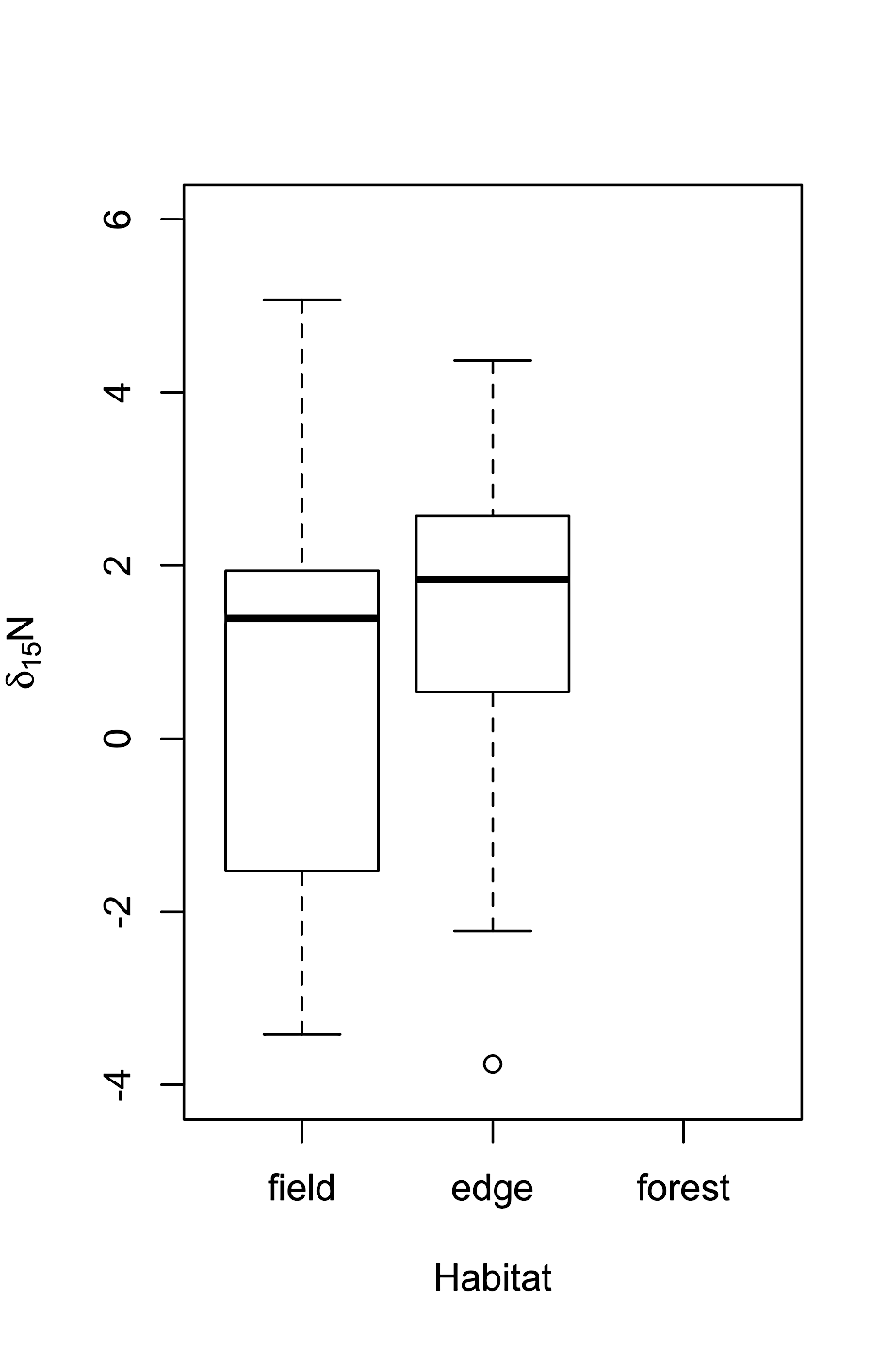

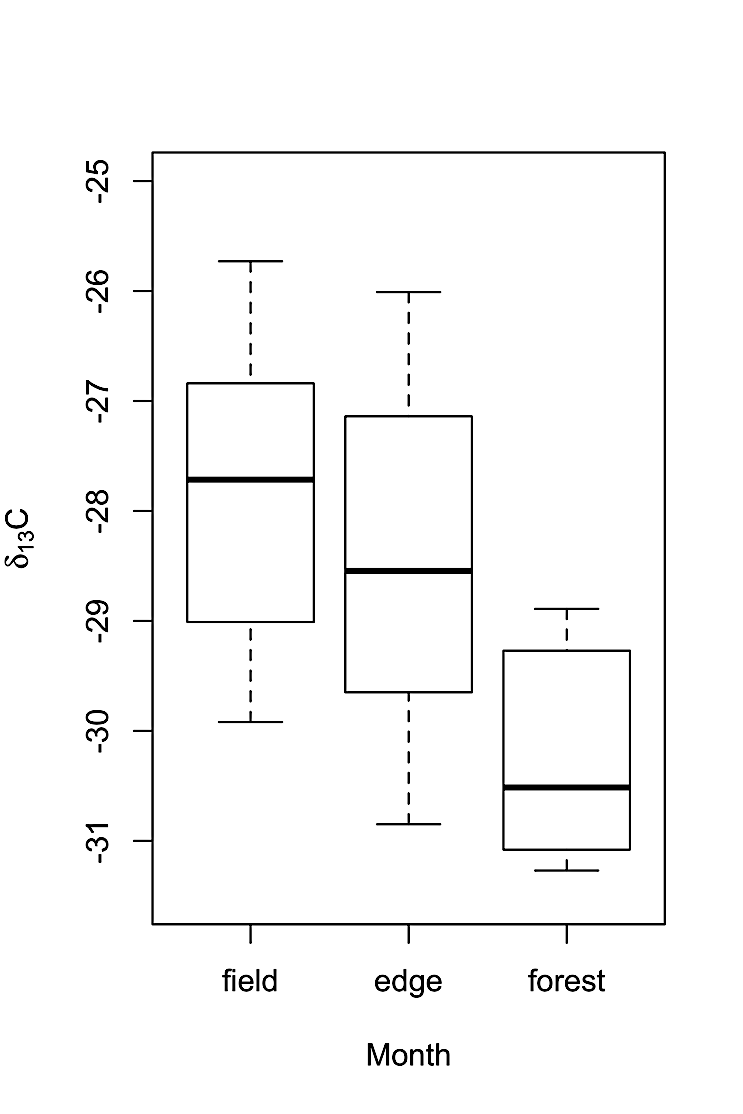

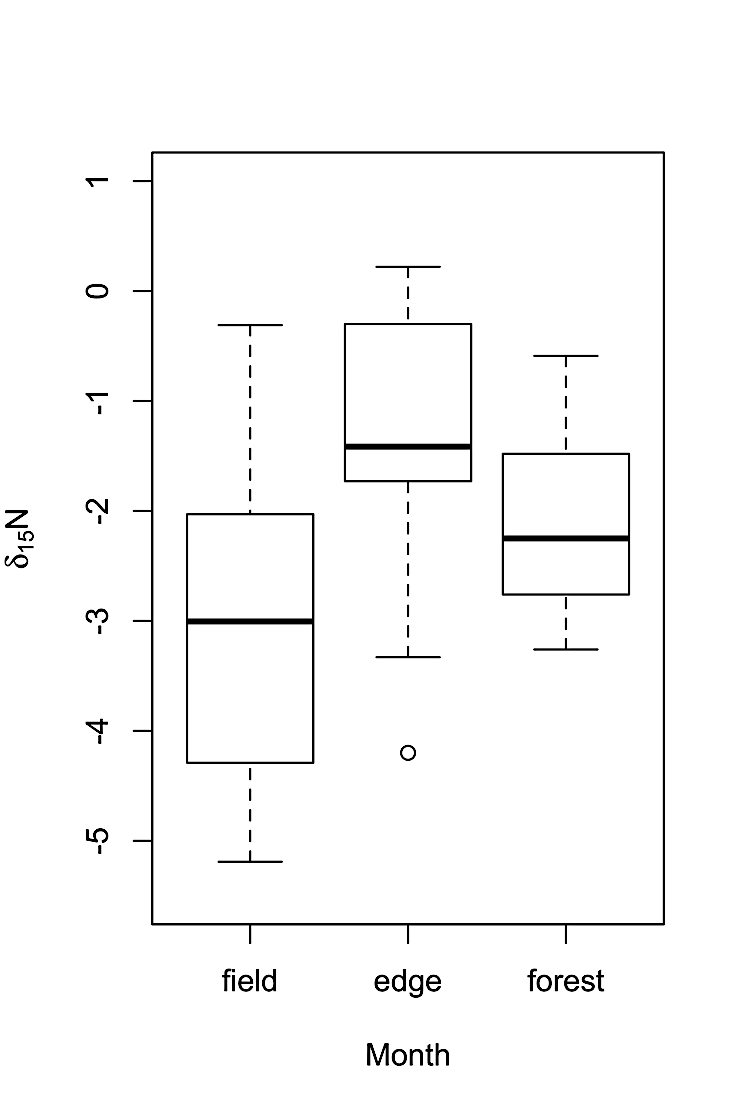


(a)

(b)

(c)

(d)

**Fig S2**. **Bee head+thorax isotope composition by taxonomic and social caste groups**.

(a) *Andrena carlini* (AprMay) are shown as dark blue squares, *A. crateaegi* (Jun) as light blue. (b) *Halictus ligatus* (Jun – Aug) are shown as dark purple squares, *H. confusus* (Jul) as light purple, (c) *Bombus* overwintered spring queens (AprMay) are shown as white squares, summer queens-of-the-year (Jul – Aug) as black, and summer workers (Jun – Aug) as gray. Bombus isotope values did not differ significantly by species. Bee ∂^13^C was lipid normalized following (McConnaughey and McRoy (1979).


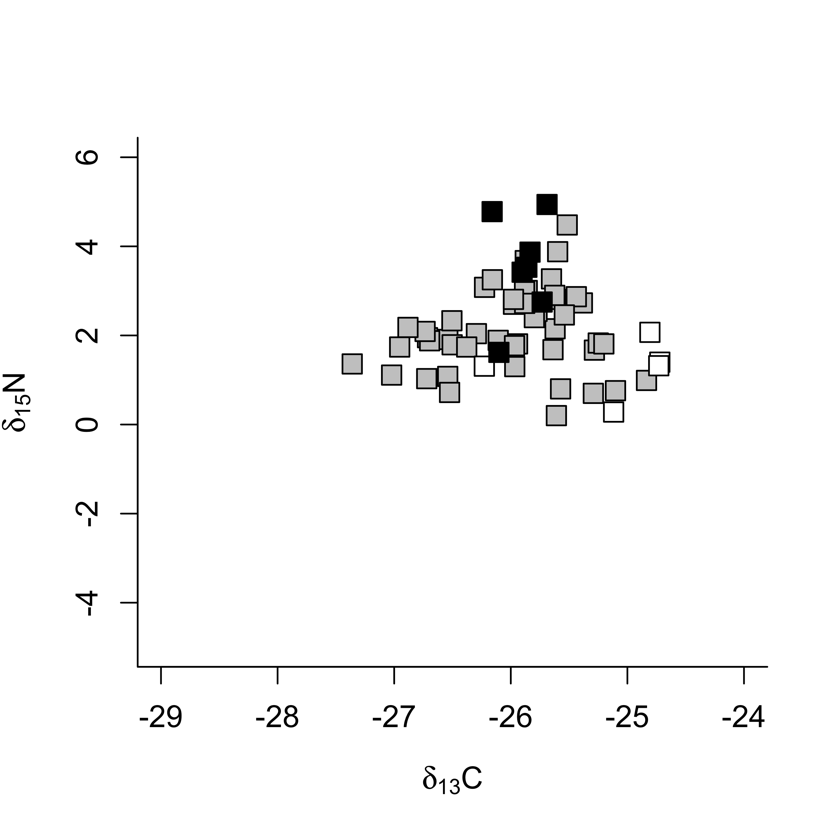

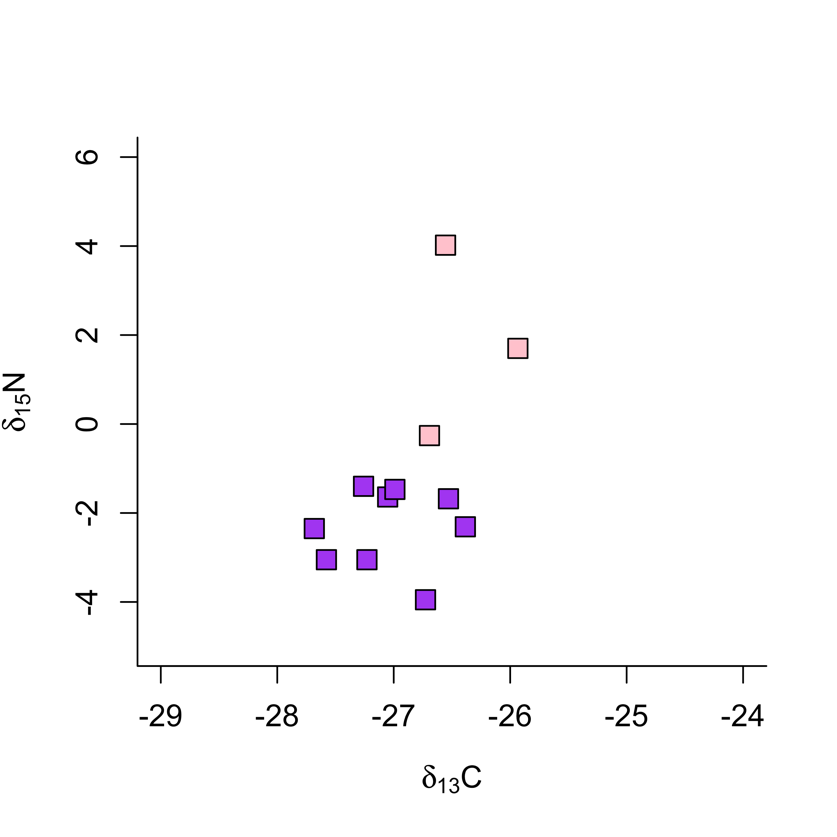

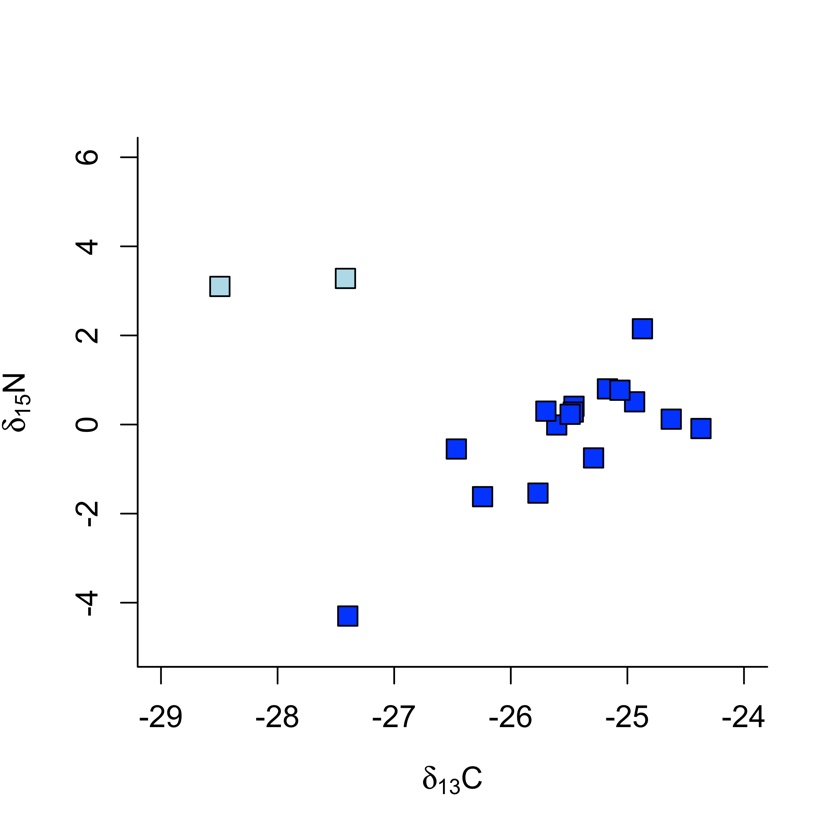


(a) *Andrena*

(b) *Halictus*

(c) *Bombus*

**Fig S3. Seasonal trends in natural abundances of C and N** **stable isotopes** **in bee head+thorax and flowers: (a) bee ∂^13^C_lipid normalized_, (b) flower ∂^13^C, (c) bee ∂^15^N, (d) flower ∂^15^N.** Samples collected at the end of April were combined with those from the beginning of May. The center line represents group median, with boxes showing upper and lower quartiles. The bars show value range. Bee sample sizes were as follows: AprMay n=21, June n=20 , July n=32, August n=16. Flower sample sizes were as follows: AprMay n=15, June n=5, July n=9, August n=3.


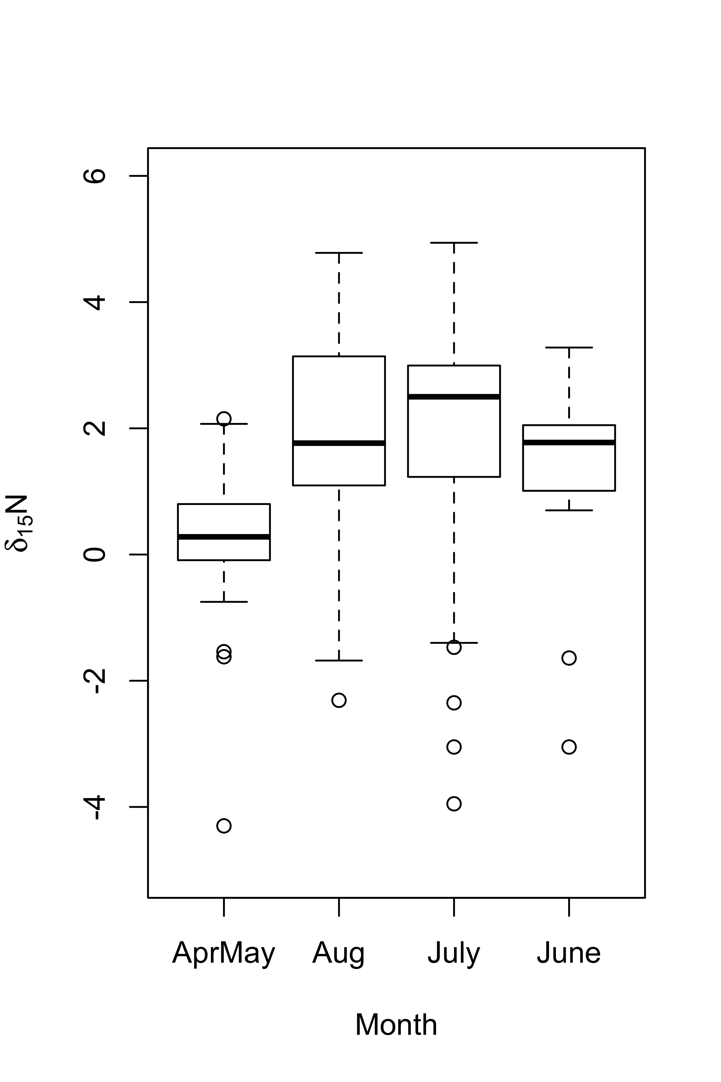

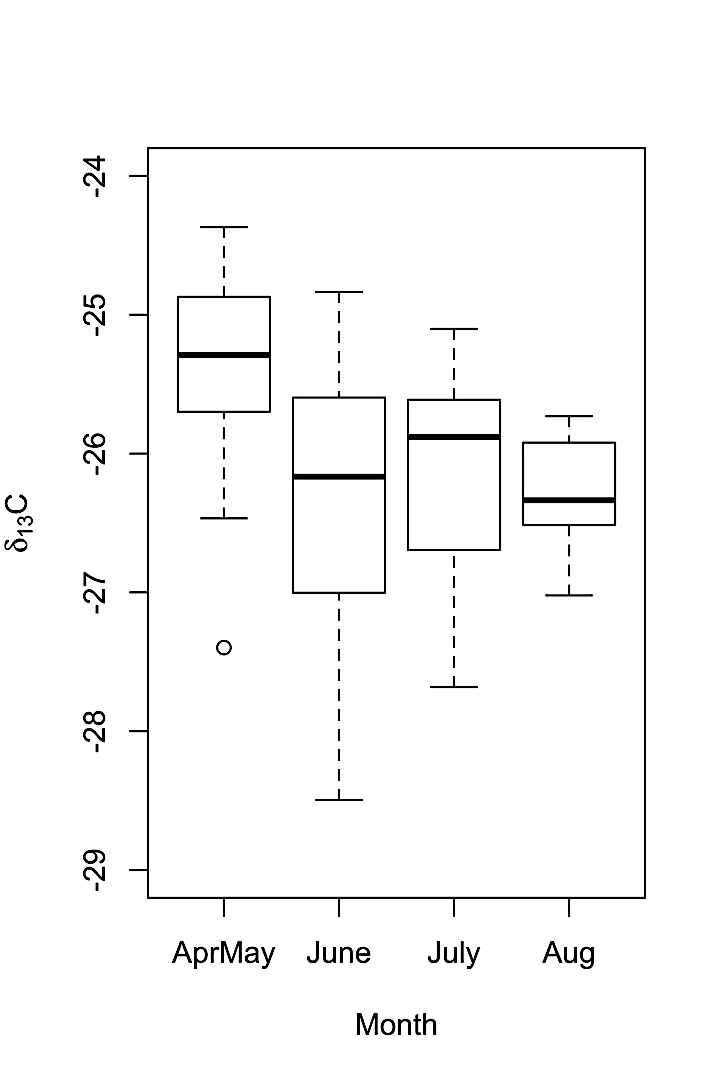


Month


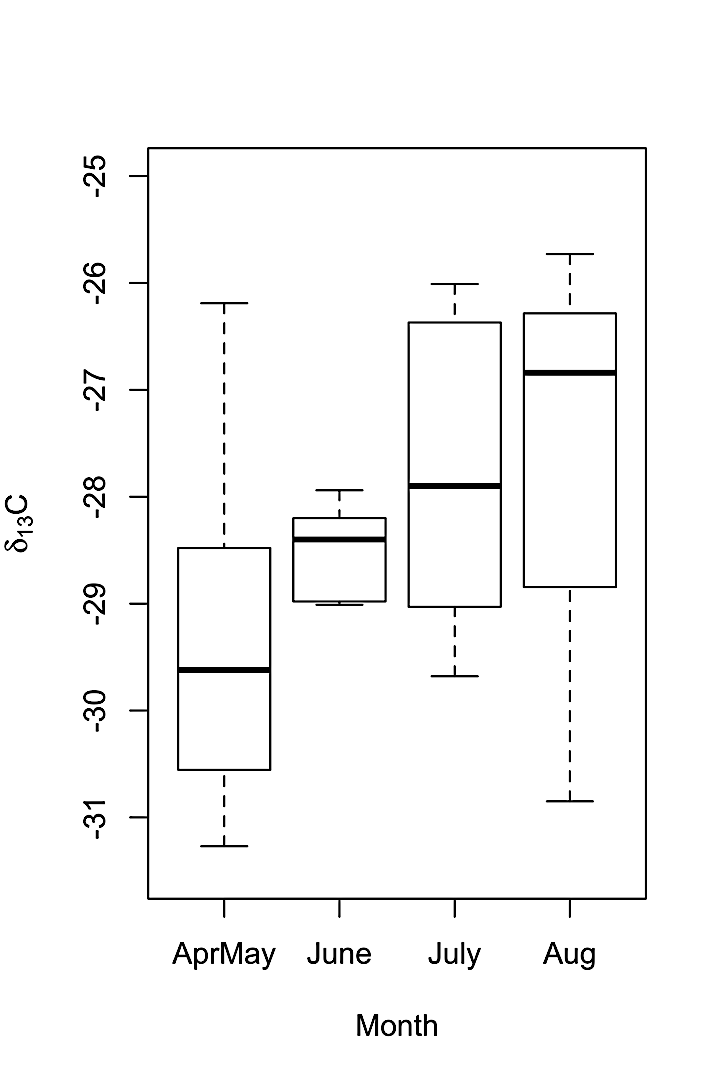


(a)

(b)


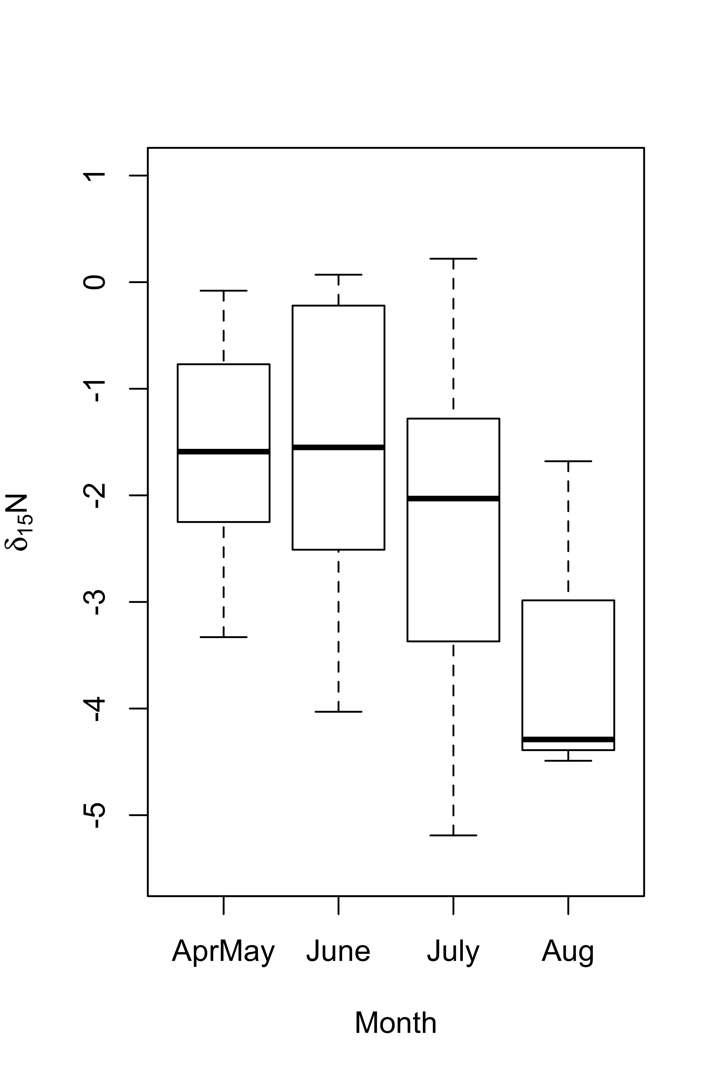


(d)

(c)
